# Combined application of anti-VEGF and anti-EGFR attenuates the growth and angiogenesis of colorectal cancer mainly through suppressing AKT and ERK signaling in mice model

**DOI:** 10.1186/s12885-016-2834-8

**Published:** 2016-10-12

**Authors:** Chenbo Ding, Longmei Li, Taoyu Yang, Xiaobo Fan, Guoqiu Wu

**Affiliations:** 1 Medical School of Southeast University, Nanjing, 210009 China; 2 Department of Immunology, Zunyi Medical University, Zunyi, 563003 China; 3 Department of Oncology, the Affiliated Hospital of Zunyi Medical University, Zunyi, 563003 China; 4 Center of Clinical Laboratory Medicine, Zhongda Hospital, Southeast University, Nanjing, 210009 China

**Keywords:** Colorectal cancer, VEGF, EGFR, Angiogenesis

## Abstract

**Background:**

Angiogenesis is generally involved during the cancer development and hematogenous metastasis. Vascular endothelial growth factor (VEGF) and epidermal growth factor receptor (EGFR) are considered to have an important role in tumor-associated angiogenesis. However, the effects of simultaneously targeting on VEGF and EGFR on the growth and angiogenesis of colorectal cancer (CRC), and its underlying mechanisms remain unknown.

**Methods:**

Immunohistochemical staining was used to detect the VEGF and EGFR expression in different CRC tissue specimens, and the correlation between VEGF/EGFR expression with the clinicopathologic features was analyzed. Cell counting kit‑8 (CCK-8) and transwell assays were used to assess the cellular proliferation and invasion of CRC cells after treated with anti-VEGF antibody and/or anti-EGFR antibody in vitro, respectively. Moreover, in vivo tumor formation was performed on nude mice model, and the tumor microvessel density (MVD) was determined by anti-CD34 staining in different groups. Finally, we evaluated the impact of anti-VEGF antibody and/or anti-EGFR antibody on the activation of downstream signaling effectors using western blot.

**Results:**

VEGF and EGFR were upregulated in CRC tissues, and their expression levels were correlated with hepatic metastasis. Blockage on VEGF or EGFR alone could inhibit the cellular proliferation and metastasis while their combination could reach a good synergism in vitro. In addition, in vivo xenograft mice model demonstrated that the tumor formation and angiogenesis were strongly suppressed by combination treatment of anti-VEGF and anti-EGFR antibodies. Besides, the combination treatment significantly reduced the activation of AKT and ERK1/2, but barely affected the activation of c-Myc, NF-κB/p65 and IκBα in CRC cells tumors. Interestingly, anti-VEGF antibody or anti-EGFR antibody alone could attenuate the phosphorylation of STAT3 as compared with negative control group, whereas the combined application not further suppressed but at least partially restored the activation of STAT3 in vivo.

**Conclusions:**

Simultaneous targeting on VEGF and EGFR does show significant inhibition on CRC tumor growth and angiogenesis in mice model, and these effects are mainly attributed to suppression of the AKT and ERK signaling pathways.

**Electronic supplementary material:**

The online version of this article (doi:10.1186/s12885-016-2834-8) contains supplementary material, which is available to authorized users.

## Background

Colorectal cancer (CRC) is one of the most common malignant tumors in the Western World, China, and other countries [1–3]. The prognosis of CRC at an early stage is favorable, as a result of improved detection of early cancer and wider implementation of radical surgery, but the prognosis of unresectable, advanced CRC is not yet satisfactory. When tumor lesions are not fully resectable or become metastatic, patients will have very limited options for target agents and conventional chemotherapy. As a result, the overall outcome of patients is barely satisfied mainly due to distant metastases formation, especially hepatic and other hematogenous metastases [4–7], since angiogenesis and hematogenous metastasis are intrinsically connected. Thus, countermeasures against tumor angiogenesis seem to be a promising strategy for improving the prognoses of these cancer patients.

Angiogenesis, the process leading to the formation of new blood vessels, plays an important role in tumor development and distant metastasis [8], and its induction is mediated by numerous angiogenic factors [9]. Among these factors, vascular endothelial growth factor (VEGF) and its receptors are the most potent molecules activating endothelial cells metastasis and increasing vascular permeability [10–12]. Inhibition of VEGF activity has been reported to suppress the proliferation of cancer cells and improve the prognosis for unresectable CRC patients [13].

In addition, epidermal growth factor receptor (EGFR), which plays an important role in tumorigenesis, is overexpressed in many types of cancers, especially in CRC [14, 15]. According to the European and US guidelines, EGFR targeting-therapy has been recommended for the treatment of metastatic colorectal cancer (mCRC) [12]. However, not all patients have a good response to anti-EGFR treatment, and there is important clinical value for identifying predictors of treatment benefit or lack thereof [16]. Resistance to anti-EGFR therapies is mediated, at least partly, through activating VEGF-mediated intracellular cascade [17, 18]. Therefore, a strategy that simultaneously targets on VEGF and EGFR agents appears to be promising in preclinical and clinical studies for the treatment of CRC.

However, very few studies have been conducted to determine the therapeutic effects of targeting both VEGF and EGFR for anti-CRC treatment. In this study, we mainly evaluated the effects of targeting both VEGF and EGFR on CRC growth and angiogenesis as well as its relative molecular mechanism using in vitro CRC cell lines and in vivo mouse model systems.

## Methods

### Patients and specimens

In this study, a total of 60 CRC tumor tissues and 30 corresponding normal tissues were prepared for immunohistochemistry assay. Normal tissues were cut at least 5 cm away from tumor margin. All the specimens were collected from patients with CRC who were treated at the Affiliated Hospital of Zunyi Medical University between May 2015 and December 2015. The study was conducted in accordance with the 1975 declaration of Helsinki and with approval from the Ethics Committee of the Affiliated Hospital of Zunyi Medical University. Written informed consent was obtained from all participants. None of the cases received neoadjuvant therapy before surgery. After surgical resection, the resected specimens were re-evaluated before the current study by two pathologists.

### Cell lines and culture conditions

The human CRC cell lines HT29, SW480, SW620 and LoVo were obtained from Cell bank of Chinese Academy of Sciences (Shanghai, China). All the cancer cells were cultured in McCoy 5A, RPMI-1640 or Leibovitz’s L-15 medium supplemented with 10 % fetal bovine serum (FBS) (HyClone, Logan, UT, USA), 100 IU/mL penicillin and 100 μg/ml streptomycin. All the cells were cultured in a humidified atmosphere of 5 % CO_2_ at 37 °C.

### Quantitative Real‑time PCR

Total RNA was extracted from cells with Trizol (Invitrogen, USA) and reverse transcribed using RT reagent Kit (TakaRa, Japan) according to the manufacturer’s instructions. Quantitative reverse transcription-PCR (qRT-PCR) analysis was performed as previous described [19]. The sequences of primers in this section are the followings: (1) VEGF: 5′-CTTGCCTTGCTGCTCTACCT-3′ (forward) and 5′-CTGCATGGTGATGTTGGACT-3′ (reverse); (2) EGFR: 5′-GAGAGGAGA ACTGCCAGAA-3′ (forward) and 5′-GTAGCATTTATGGAGAGTG-3′ (reverse); (3) GAPDH: 5′-GAAGGTGAAGGTCGGAGTC-3′ (forward) and 5′-GAAGATGGTGATGGGATTTC-3′ (reverse). GAPDH was used as an internal control.

### Western blot analysis

Western blot analysis was performed as previous described [19]. The following commercial antibodies were used in this study: VEGF, EGFR, phospho-c-Myc, total c-Myc, phospho-NF-κB/p65, total NF-κB/p65, phospho-IκBα and total IκBα (Abcam, UK), phospho-AKT, total AKT, phospho-STAT3, total STAT3, phospho-ERK1/2 and total ERK1/2 (Invitrogen, USA), GAPDH and β-actin (Immunology Consultants Laboratory, USA).

### Cell counting kit‑8 assay

The Cell Counting Kit-8 (CCK-8) assay kit (Dojindo, Kumamoto, Japan) was used to determine the impact of anti-human VEGF mAb and/or anti-human EGFR mAb on cell proliferation. The concentrations of anti-VEGF or anti-EGFR used in these assays are as following: 0, 0.25, 0.5, 1 and 2 μg/ml. Cells were plated in 96-well plates at a density of 1 × 10^4^ cells per well for 48 h. 10 μl CCK-8 solution was added to the cells for 2.5 h at 37 °C, and the viability of the cells was measured at 450 nm using an ELISA reader (BioTek, Winooski, VT, USA) according to the manufacturer’s instructions. For each experimental condition, 3 wells were used, and the experiments were repeated 3 times.

### Invasion assay

Invasion assays were performed as reported [20]. Transwell invasion assays were performed in Corning Matrigel invasion chamber containing an 8 μm pore-size polycarbonate membrane with a uniform layer of BD Matrigel basement membrane matrix (BD Biosciences, USA). Three independent experiments were performed with triplicate wells.

### in vivo tumor xenograft model

Female BALB/C nude mice (5–6 weeks old) were used for xenograft studies. 2 × 10^6^ of control and experimental cells suspended in phosphate-buffered saline (PBS) were injected subcutaneously into the right armpit of mice (six mice each group). Four groups of mice were tested. Group A was injected with CRC cells (SW620/LoVo) and non-specific mouse IgG. Group B was injected with CRC cells and the anti-mouse VEGF mAb (10 μg), which could react with human and mouse source VEGF protein. Group C was injected with CRC cells and the anti-mouse EGFR mAb (10 μg), which could react with human and mouse source EGFR protein. Group D was injected with CRC cells and the anti-mouse VEGF mAb (10 μg) and anti-mouse EGFR mAb (10 μg). Tumor volume was determined by external measurement according to the formula (d^2^ × D)/2 [21]. Mice were sacrificed after 35 days, and tumors were harvested, weighted and examined histologically.

### Immunohistochemical studies

Immunohistochemical assay for paraffin-embedded tissues were performed as reported [19, 20]. The evaluation principle was quantified based on the immunoreactive score (IRS), which was calculated as a product of staining intensity (SI) and percentage of positive cells (PP). SI is determined as follows: no staining (score 0), light yellow (score 1), buffy (score 2) and brown (score 3). PP is determined as follows: less than 5 % (score 0), 6 %–25 % (score 1), 26 %–50 % (score 2), 51 %–75 % (score 3) and >76 % (score 4). By multiplying SI and PP, the final weighed expression score was ranged from 0 to 12. Five random fields in each section were selected for the evaluation. The sections scoring at least 3 points in our study were indicating positive protein expression.

### Quantification of microvessel density

Tumor MVD was determined as described [22]. The slides were examined under × 100 magnication to identify the highest vascular density area within the tumor, and one field magnified 200-fold in each of five vascularized areas was counted. The average of the five areas was recorded as the MVD level of this case. Any brown-staining endothelial cell or endothelial cell cluster that was clearly separate from adjacent microvessels, tumor cells, and other connective tissue elements was considered as a single, countable microvessel.

### IL6 ELISA

Supernatants collected from CRC cell xenografts were assayed by the IL6 ELISA Kit (Invitrogen) according to the manufacturer’s instructions. Experiments were performed in duplicates.

### Statistical analysis

All values were represented as the mean ± SEM from at least three independent experiments. Clinical correlative studies were performed by Pearson’s *χ* ^2^-test using SPSS19.0 software system. Student’s *t*-test for two groups or one-way analysis of variance (ANOVA) for three or more groups were performed to evaluate the statistical significance by using GraphPad Prism 5 software. *P* values less than 0.05 were considered statistically significant.

## Results

### Clinical significance of VEGF/EGFR expression in CRC tissues

**Fig. 1:**
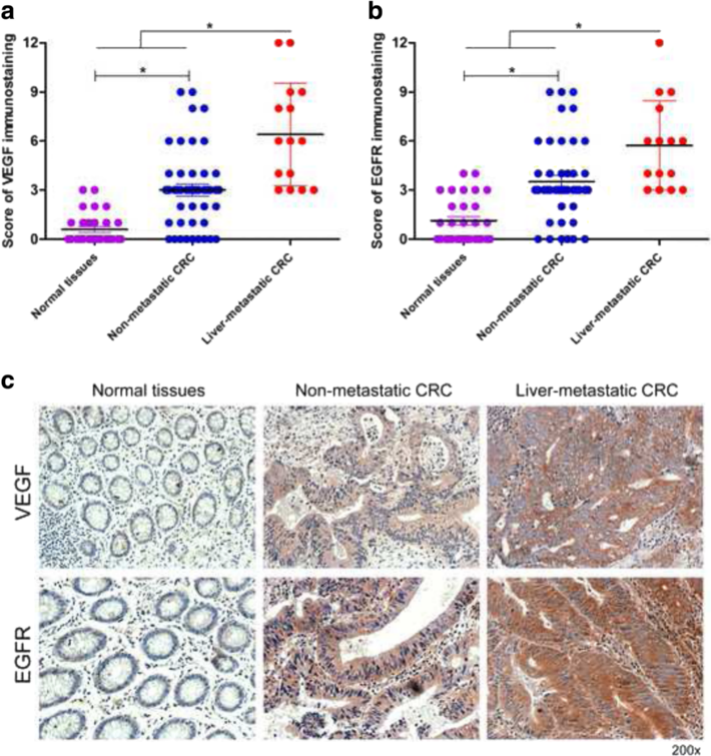
VEGF and EGFR expression are significantly upregulated in liver-metastatic CRC tissues. **a** Results of VEGF staining were evaluated by the staining scores. **b** Results of EGFR staining were evaluated by the staining scores. **c** Immunohistochemistry analysis of VEGF and EGFR expression in different colorectal tissues. *P < 0.05

It has been widely recognized that VEGF and EGFR are overexpressed in CRC tissues. In this study, we also detected the expression of VEGF and EGFR in different colorectal tissues. The VEGF and EGFR expression levels were evidently higher in liver-metastatic CRC samples than that in non-metastatic CRC samples or noncancerous samples (Fig. 1a and b). The VEGF and EGFR expression levels in non-metastatic CRC tissues were also higher than that in normal tissues (Fig. 1a and b). In addition, the results of immunohistochemical staining showed that positive signals of VEGF and EGFR were mainly occurred in the cell membrane and cytoplasm (Fig. 1c).

To further identify the clinical importance of VEGF/EGFR in CRC, we analyzed the correlationship between the VEGF/EGFR protein level with clinicopathological characteristics, including age, gender, tumor size, histology, tumor location, differentiation status, hepatic metastasis and TNM stage. Strikingly, VEGF expression was evidently correlated with tumor size, hepatic metastasis and TNM stage (Table 1). However, no relationship was found between the VEGF expression and other clinicopathological characteristics including age, gender, histology, tumor location and differentiation status (Table 1). In addition, EGFR expression was evidently correlated with tumor size, differentiation status, hepatic metastasis and TNM stage (Table 1). However, no relationship was found between the EGFR expression and other clinicopathological characteristics including age, gender, histology, and tumor location (Table 1). Taken together, these data strongly indicated that VEGF and EGFR were positively correlated with the metastasis of CRC.

**Table 1:** Clinicopathologic factors and VEGF/EGFR expression in 60 CRC patients

Characteristics	Total (N)	VEGF expression		*P*-value	EGFR expression		*P*-value
		Positive	Negative		Positive	Negative	
Age (years)				0.832			0.406
> 60	34	24	10		29	5	
≤ 60	26	19	7		20	6	
Gender				0.761			0.128
Male	37	26	11		28	9	
Female	23	17	6		21	2	
Tumor size (cm)				0.043*			0.018*
> 5	40	32	8		36	4	
≤ 5	20	11	9		13	7	
Histology				0.950			0.827
Tubular	42	30	12		34	8	
Mucinous/Papillary	18	13	5		15	3	
Tumor location				0.890			0.982
Colon	22	16	6		18	4	
Rectal	38	27	11		31	7	
Differentiation status				0.101			0.027*
Well/Moderate	44	29	15		33	11	
Poor	16	14	2		16	0	
Hepatic metastasis				0.005*			0.034*
Absent	45	28	17		34	11	
Present	15	15	0		15	0	
TNM stage				0.037*			0.012*
I-II	41	26	15		30	11	
III-IV	19	17	2		19	0	

*Note*: *P < 0.05

### VEGF/EGFR expression in CRC cell lines

**Fig. 2:**
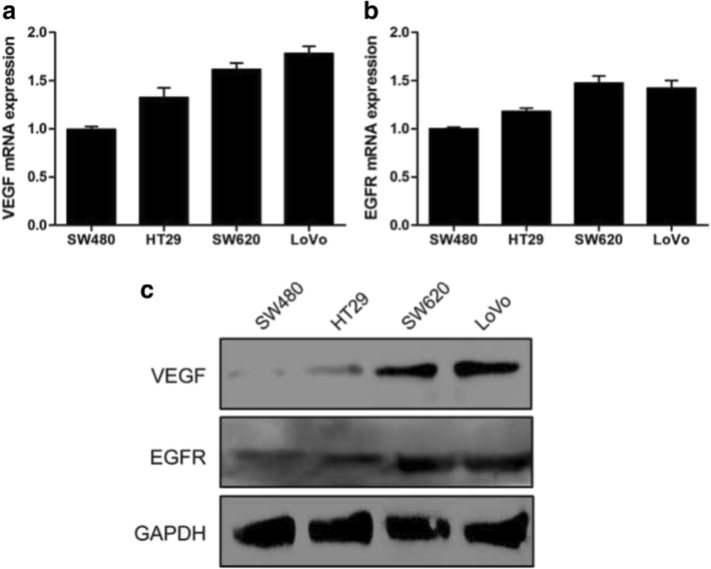
Expression of VEGF and EGFR in CRC cell lines. **a** Expression of VEGF in four human CRC cell lines was detected by qRT-PCR. **b** Expression of EGFR in four human CRC cell lines was detected by qRT-PCR. **c** Western blot analysis of VEGF and EGFR expression in different CRC cell lines

Furthermore, we detected the VEGF/EGFR expression in CRC cell lines and found that VEGF/EGFR expression in the highly invasive CRC cell lines (SW620 and LoVo) were evidently up-regulated than those in the minimally metastatic CRC cell lines (SW480 and HT29) (Fig. 2).

### Effects of combination anti-VEGF mAb and anti-EGFR mAb on CRC cells growth and invasion in vitro

**Fig. 3:**
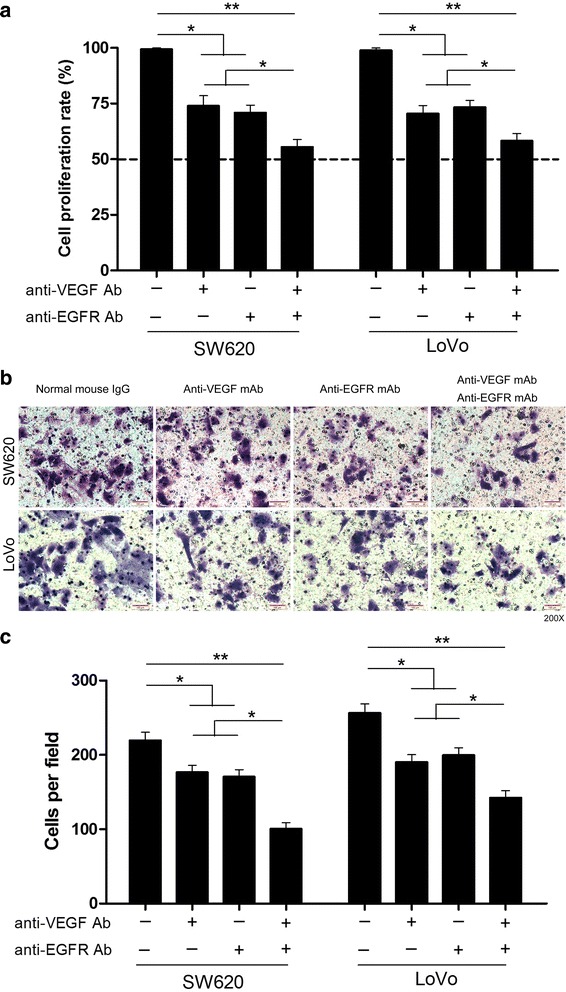
Combined application of anti-VEGF and anti-EGFR antibodies suppresses the proliferation and invasion of CRC cells in vitro. **a** The proliferation rate of SW620 and LoVo cells were analyzed by CCK-8 assay in different groups. **b** Invasion assay of SW620 and LoVo cells in different groups. **c** Invasion of SW620 and LoVo cells were quantitatively analyzed in different groups. Columns are the average of three independent experiments ± SEM. *P < 0.05; **P < 0.01

In order to confirm the role of anti-VEGF mAb (monoclonal antibody) or anti-EGFR mAb on CRC cells growth in vitro, SW620 and LoVo cells were treated with different concentrations of anti-VEGF mAb or anti-EGFR mAb. Cell counting kit‑8 (CCK-8) assay kit was used to detect proliferation activity of these cells. The results showed that anti-VEGF mAb or anti-EGFR mAb could independently prohibit the cell proliferation in a concentration dependent manner (Additional file 1: Figure S1). Considered facilitately observed the experimental results, we chose moderate anti-VEGF mAb or anti-EGFR mAb concentration (0.5 μg/ml) to explore the proliferation and invasion of CRC cells in vitro. As shown in Fig. 3a, the proliferation of SW620/LoVo cells was obviously inhibited in the presence of both anti-VEGF mAb and anti-EGFR mAb, compared with the presence of anti-VEGF mAb or anti-EGFR mAb alone. Transwell assay identified that the invasion of SW620/LoVo cells was suppressed in the presence of anti-VEGF mAb or anti-EGFR mAb alone, compared with negative control group (Fig. 3b and c). When both anti-VEGF mAb and anti-EGFR mAb were present, the mobility of these cells was further reduced (Fig. 3b and c). These results revealed that both anti-VEGF mAb and anti-EGFR mAb could suppress growth and metastasis of CRC cells in culture.

### Effect of anti-VEGF mAb and anti-EGFR mAb on CRC cells tumorigenicity in vivo

**Fig. 4:**
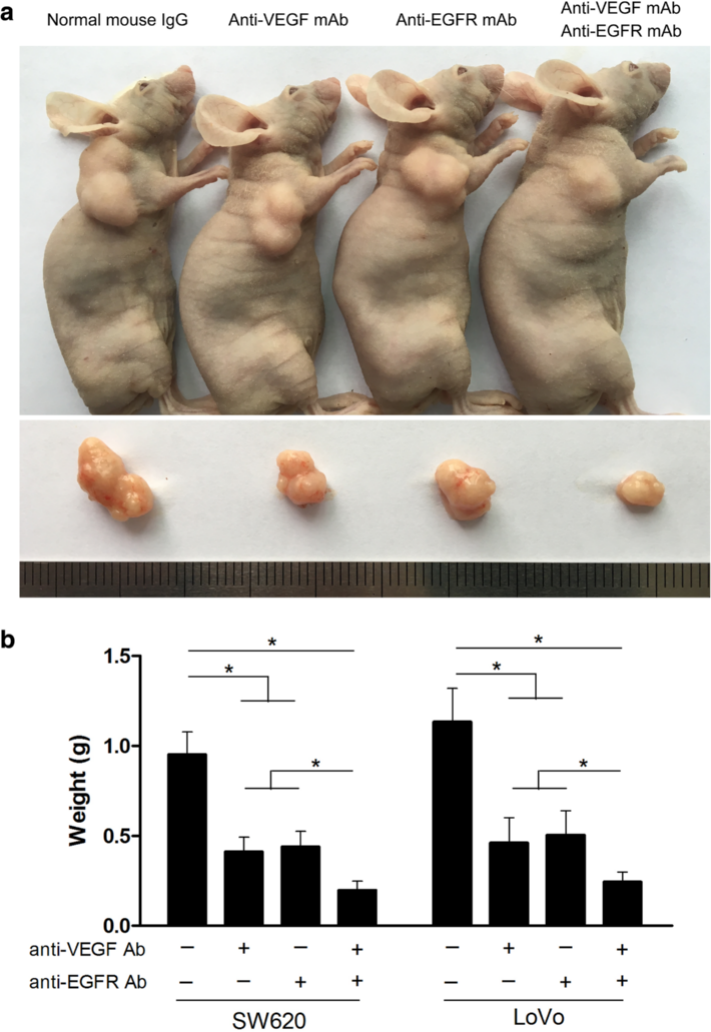
Suppression of CRC cells tumorigenicity by anti-VEGF and anti-EGFR antibodies in vivo. **a** Representative photographs of tumor formation in mice in response to SW620 cells. **b** Five weeks later, the tumors were resected and weighted. The weight analyzed with Student’s *t*-test. Data represent means ± SEM. *P < 0.05

Cultured SW620 cells were subcutaneously injected in mice, and tumor formation was observed 35 days after injection (Fig. 4a). In addition, tumor weight was measured in these groups. As a result (Fig. 4b), the average tumor weight of SW620 cells in the presence of both anti-VEGF mAb and anti-EGFR mAb was 0.198 ± 0.022 g, which was significantly lower (*P* < 0.05) than that of mice inoculated with anti-VEGF mAb (0.412 ± 0.036 g), anti-EGFR mAb (0.440 ± 0.038 g), and negative control group (0.952 ± 0.056 g). When LoVo cells were injected with non-specific mouse IgG, the average tumor weight was 1.134 ± 0.083 g. It was 0.462 ± 0.062 g in the presence of anti-VEGF mAb, 0.506 ± 0.059 g in the presence of anti-EGFR mAb, and 0.244 ± 0.025 g in the presence of both anti-VEGF and anti-EGFR antibodies (Fig. 4b). These results suggested that both anti-VEGF mAb and anti-EGFR mAb could inhibit CRC cells tumorigenicity in vivo.

### Suppression of tumor angiogenesis by CRC cells after application of anti-VEGF mAb and anti-EGFR mAb

**Fig. 5:**
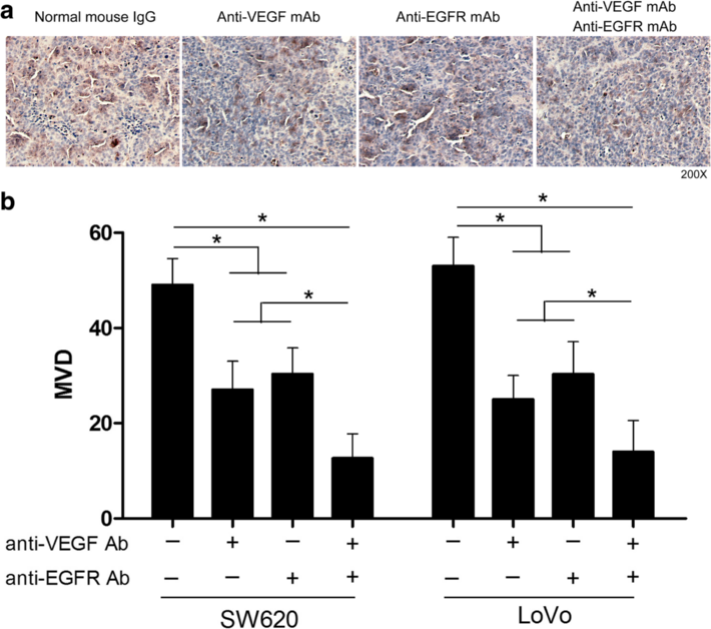
Suppression of CRC cells tumor angiogenesis by anti-VEGF and anti-EGFR antibodies. **a** Representative photographs of anti-CD34 staining in SW620 cells tumors. **b** The numbers of positively CD34 stained cells in subcutaneous SW620 and LoVo cells tumors. The data are representative of at least three different experiments ± SEM. *P < 0.05

Accordingly, the amount of microvessel density (MVD) determined using anti-CD34 mAb immunostaining in the same mouse tumors (Fig. 5a and b). The number of positive cells in SW620 tumors with non-specific mouse IgG was 49.00 ± 3.22 per field-of-view, 27.00 ± 3.46, 30.33 ± 3.18 per field-of-view in the presence of anti-VEGF mAb or anti-EGFR mAb, and 12.67 ± 2.96 in the presence of both antibodies. In the LoVo tumors, there were 53.00 ± 3.46 positive cells per field-of-view in negative control group. 25.00 ± 2.89, 30.33 ± 3.93 cells were observed in the presence of anti-VEGF mAb or anti-EGFR mAb, and 14.00 ± 3.79 cells were observed in the presence of both antibodies (Fig. 5b). These results indicated that both anti-VEGF and anti-EGFR antibodies could reduce tumor angiogenesis.

### The activity of VEGF and EGFR-dependent signaling in CRC cells tumors after application of anti-VEGF and anti-EGFR antibodies

**Fig. 6:**
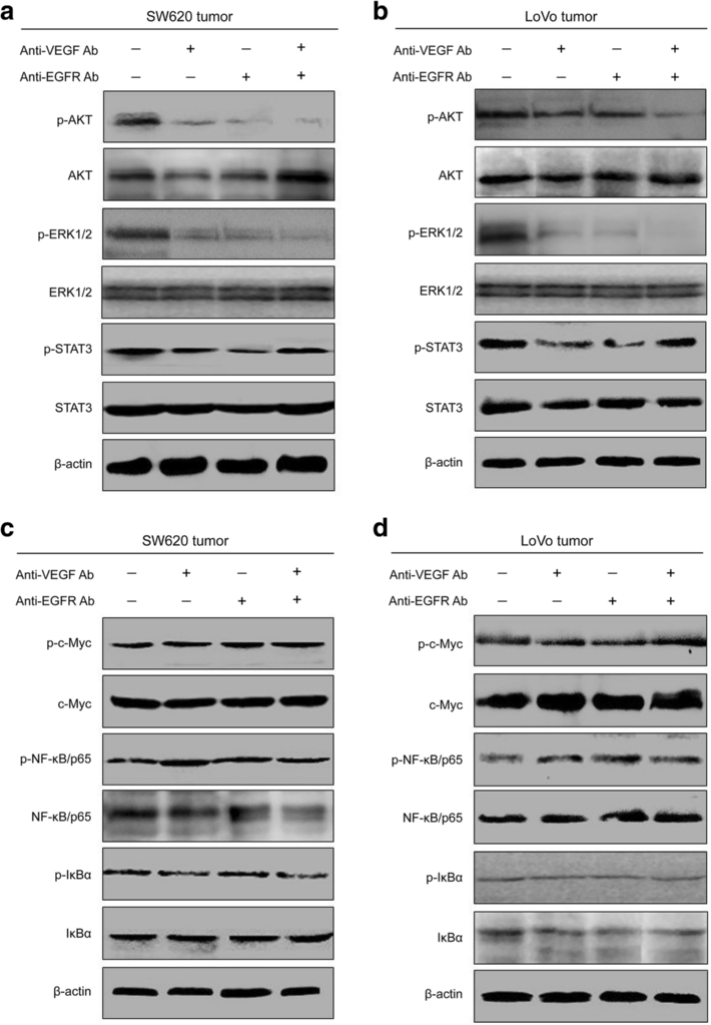
Combined application of anti-VEGF and anti-EGFR antibodies attenuates the activation of AKT and ERK, but not STAT3, c-Myc and NF-κB in vivo. **a** Western blot analysis was performed to determine the activation of AKT, ERK1/2 and STAT3 in different SW620 cells tumors. **b** Western blot assay of different LoVo cells tumors (one clone, **a**). **c** Western blot analysis was performed to determine the activation of c-Myc, NF-κB/p65 and IκBα in different SW620 cells tumors. **d** Western blot assay of different LoVo cells tumors (one clone, **c**)

As both VEGF and EGFR can activate phosphatidylinositol 3-kinase (PI3K), mitogen activated protein kinase (MAPK) and janus kinase (JAK) signaling pathways [23–26], we examined the downstream effectors, AKT, extracellular signal-regulated kinase (ERK) and signal transducer and activator of transcription 3 (STAT3), respectively. As expected, VEGF or EGFR inhibition by mAb downregulated the phosphorylation of AKT, ERK1/2 and STAT3 as compared with negative control group (Fig. 6a, b and Additional file 2: Figure S2A, B). In addition, the phosphorylation of AKT and ERK1/2 was further reduced in the presence of both anti-VEGF mAb and anti-EGFR mAb as compared with anti-VEGF mAb or anti-EGFR mAb alone (Fig. 6a, b and Additional file 2: Figure S2A, B). Unfortunately, when combined treatment with anti-EGFR and anti-VEGF antibodies, the phosphorylation of STAT3 was not further suppressed, but at least, partially restored (Fig. 6a, b and Additional file 2: Figure S2A, B). It has been widely recognized that STAT3 signaling pathway links inflammation to cell transformation, and STAT3 activation is dependent on IL6 levels [27]. To explore whether IL6 expression associates with the activation of STAT3 signaling, we detected the expression of IL6 in the same mouse tumors. The levels of IL6 were slightly decreased in the presence of anti-VEGF mAb or anti-EGFR mAb alone, compared with negative control group. However, when both anti-VEGF mAb and anti-EGFR mAb were present, IL6 levels were significantly up-regulated in CRC cell tumors (Additional file 3: Figure S3). These results suggested that anti-VEGF and anti-EGFR antibodies could attenuate PI3K and ERK signaling, but not IL6/STAT3 signaling in CRC cell tumors.

Notably, other signaling effectors, such as c-Myc oncogene [28] and nuclear factor kappa B (NF-κB) [29] are frequently reported to involve in the development of many types of tumors. In addition, IκBα functions as an inhibitor of NF-κB, which interacts with p65 to form an inactive NF-κB/IκBα complex, and then inhibits the activation of NF-κB signaling pathway [30, 31]. In order to determine whether anti-VEGF and anti-EGFR antibodies could suppress the activation of these signaling effectors, we examined the expression of c-Myc, NF-κB/p65 and IκBα in CRC cell tumors by western blot. We found that the activation of phospho-c-Myc, phospho-NF-κB/p65 and phospho-IκBα was not marked increased or decreased in the presence of anti-VEGF antibody or anti-EGFR antibody compared with the control group (Fig. 6c, d and Additional file 2: Figure S2C, D). Moreover, when both anti-VEGF and anti-EGFR antibodies were present, there were also no significant differences in the activation of these factors (Fig. 6c, d and Additional file 2: Figure S2C, D). Taken together, these findings indicated that anti-VEGF and anti-EGFR antibodies couldn’t sufficient to inhibiting the activation of c-Myc and NF-κB.

## Discussion

It has been demonstrated that overexpression of VEGF/EGFR is correlated with the progression and metastasis of CRC [32–34]. Despite that previous studies found the suppressing role of anti-VEGF/EGFR antibody on CRC development [35, 36], the potential effect of combination anti-VEGF and anti-EGFR antibodies on CRC growth and angiogenesis remains little known. In this study, we have shown that both increased VEGF and EGFR were associated with hepatic metastases in CRC. Additionally, we found that anti-VEGF and EGFR antibodies could not only reduce CRC cells proliferation and invasion in vitro, but also inhibit the tumor growth and angiogenesis in vivo mainly through prohibiting the activation of AKT and ERK signaling pathways. However, monoclonal antibodies targeting VEGF and EGFR may be un-sufficient to controlling the activity of other signaling pathways such as IL6/STAT3 signaling, which may exemplify the underlying mechanism of anti-tumor resistance.

Animal studies have manifested that inhibition of VEGF suppresses both tumor angiogenesis and tumor growth in vivo [37, 38]. Preclinical and clinical studies also suggest that inhibition of VEGF pathway causes direct and rapid changes to the tumor vasculature, and improves the overall survival rate of mCRC patients [39, 40]. In addition, there is accumulating evidence suggesting that before the selection of anti-VEGF agents, anti-EGFR agents deliver their maximum efficacy in mCRC patients when given early in the treatment strategy [41]. Of note, many studies have indicated that EGFR has a potent effect on tumor-associated angiogenesis and combined treatment with anti-EGFR and anti-VEGF antibodies have at least additive antitumor activity [42, 43]. Importantly, clinical trials have also produced promising data: combining the anti-VEGF monoclonal antibody bevacizumab with the anti-EGFR antibody cetuximab or the EGFR tyrosine kinase inhibitor erlotinib increases benefit compared with either of these anti-EGFR agents alone or combined with chemotherapy [44]. In this study, we found that the proliferation and invasion of CRC cells were obviously inhibited in the presence of both anti-VEGF and anti-EGFR antibodies in culture. Furthermore, when anti-EGFR combined with anti-VEGF treatment, the tumor growth and angiogenesis were significantly suppressed compared with other groups. These findings provided new evidence supporting the collaboration of anti-VEGF and anti-EGFR antibodies in inhibiting tumor growth and angiogenesis.

The mitogen-activated extracellular signal-regulated kinase (MEK)/ERK and PI3K/AKT signaling pathways are often concurrently activated in CRC, which are associated with the progression, metastasis and drug resistance of CRC [45–47]. VEGF and EGFR are known to function as two upstream effectors of the PI3K and MAPK pathways [23, 24]. In this study, we demonstrated that anti-VEGF antibody cooperated with anti-EGFR antibody in suppressing the phosphorylation of AKT and ERK1/2 in nude mouse model. In addition, STAT3 is persistently activated in many human cancers during cancer development and progression [48]. Interestingly, we found that anti-VEGF antibody or anti-EGFR antibody alone could attenuate the activation of STAT3, but simultaneous targeting of both VEGF and EGFR partially restored the phosphorylation of STAT3 in vivo. Although VEGF and EGFR can activate JAK/STAT3 signaling pathway, a variety of extracellular stimuli especially IL6 expression, is necessary for the activation of STAT3 signaling [25–27]. Hence, we detected the expression of IL6 in CRC cell tumors, and found that the trend of IL6 levels were consistent with that of STAT3 activation in the same tumors. One plausible explanation is that when anti-VEGF mAb or anti-EGFR mAb alone inhibits tumor growth, it also slightly decreases the expression of IL6 because of destruction of some tumor cells, while anti-VEGF and anti-EGFR antibodies simultaneously suppress tumorigenesis, the surviving tumor cells increase IL6 levels to escape the killing effect. Of note, it has been widely recognized that inhibition of JAK/STAT3 signaling is participated in chemotherapeutic sensitivity of CRC patients [49, 50]. These data suggested that combined application of anti-VEGF and anti-EGFR antibodies could sufficient suppress the activation of AKT and ERK signaling, but not IL6/STAT3 signaling pathway, and may indicate the underlying mechanism of chemotherapeutic resistance.

Of further interest, we examined the impact of both anti-VEGF and anti-EGFR antibodies on the activation of c-Myc and NF-κB in CRC cell tumors. Unfortunately, we found no significant differences between the phosphorylation of c-Myc, NF-κB/p65, IκBα, and the application of anti-VEGF antibody and/or anti-EGFR antibody in vivo. Despite that previous studies suggested the enigmatic regulation mechanisms of c-Myc and NF-κB activation, the role of activated c-Myc and NF-κB in tumor drug resistance is very affirmative [51, 52]. Of note, persistent activation of STAT3 signaling promotes uncontrolled growth and survival through dysregulation of gene expression including c-Myc, and thereby contributes to oncogenesis [53]. In addition, constitutive and persistent NF-κB activation in cancer cells is partly dependent on STAT3 status [54]. These findings and our results implied a plausible hypothesis that the invalid effect of both anti-VEGF and anti-EGFR antibodies on the activity of c-Myc and NF-κB is partly attributed to the STAT3 status.

Progression-free and overall survival in patients with mCRC was improved greatly by the addition of anti-VEGF and/or anti-EGFR to standard chemotherapy, in either first- or second-line treatment. [12, 55]. However, several clinical results have suggested that VEGF and EGFR combinatory therapy do not improve the overall prognosis in CRC [56]. In the present work, we convincingly showed that simultaneous targeting of both VEGF and EGFR could further suppress the proliferation and invasion of CRC cells in vitro, and further inhibit CRC cell tumor growth and angiogenesis through downregulation of AKT and ERK signaling in vivo. Although our data is not in line with clinical results, the STAT3 status, at least, may partly explain the inefficiencies of VEGF/EGFR co-inhibition in clinical trial.

## Conclusions

In conclusion, this study demonstrates that combined application of anti-VEGF and anti-EGFR antibodies could inhibit CRC growth and angiogenesis mainly by suppressing AKT and ERK signaling pathways in mice model. However, other molecular targets including STAT3, c-Myc and NF-κB may contribute to enhance the risk of drug-resistance to chemotherapy targeting VEGF and EGFR.

## Additional files

Additional file 1: Figure S1. Effect of different anti-VEGF mAb or anti-EGFR mAb concentration on the proliferation of SW620 and LoVo cells in vitro. **A** The proliferation rate of SW620 and LoVo cells were analyzed by CCK-8 assay in different anti-VEGF mAb concentration. **B** The proliferation rate of SW620 and LoVo cells were analyzed by CCK-8 assay in different anti-EGFR mAb concentration. (TIF 1121 kb)
Additional file 2: Figure S2. Combined application of anti-VEGF and anti-EGFR antibodies inhibits AKT and ERK signaling pathways in mice model. **A** The expression of p-AKT, p-ERK1/2 and p-STAT3 were quantitatively analyzed in different SW620 cells tumors. **B** Western blot assay of different LoVo cells tumors (one clone, **A**). **C** The expression of p-c-Myc, p-NF-κB/p65 and p-IκBα were quantitatively analyzed in different SW620 cells tumors. **D** Western blot assay of different LoVo cells tumors (one clone, **C**). The data are representative of at least three different experiments ± SEM. **P* < 0.05 (TIF 1285 kb)
Additional file 3: Figure S3. The levels of IL6 in CRC cell tumors. **A** The expression of IL6 was quantitatively analyzed in different SW620 cells tumors. **B** ELISA assay of different LoVo cells tumors (one clone, **A**). The data are representative of at least three different experiments ± SEM. NS: No statistical significance; **P* < 0.05 (TIF 1783 kb)
