# Supplementary figures and images for: Combined application of anti-VEGF and anti-EGFR attenuates the growth and angiogenesis of colorectal cancer mainly through suppressing AKT and ERK signaling in mice model
Source: BMC Cancer. 2016 Oct 12;16:791. doi: 10.1186/s12885-016-2834-8 (PMC5059930; doi:10.1186/s12885-016-2834-8)

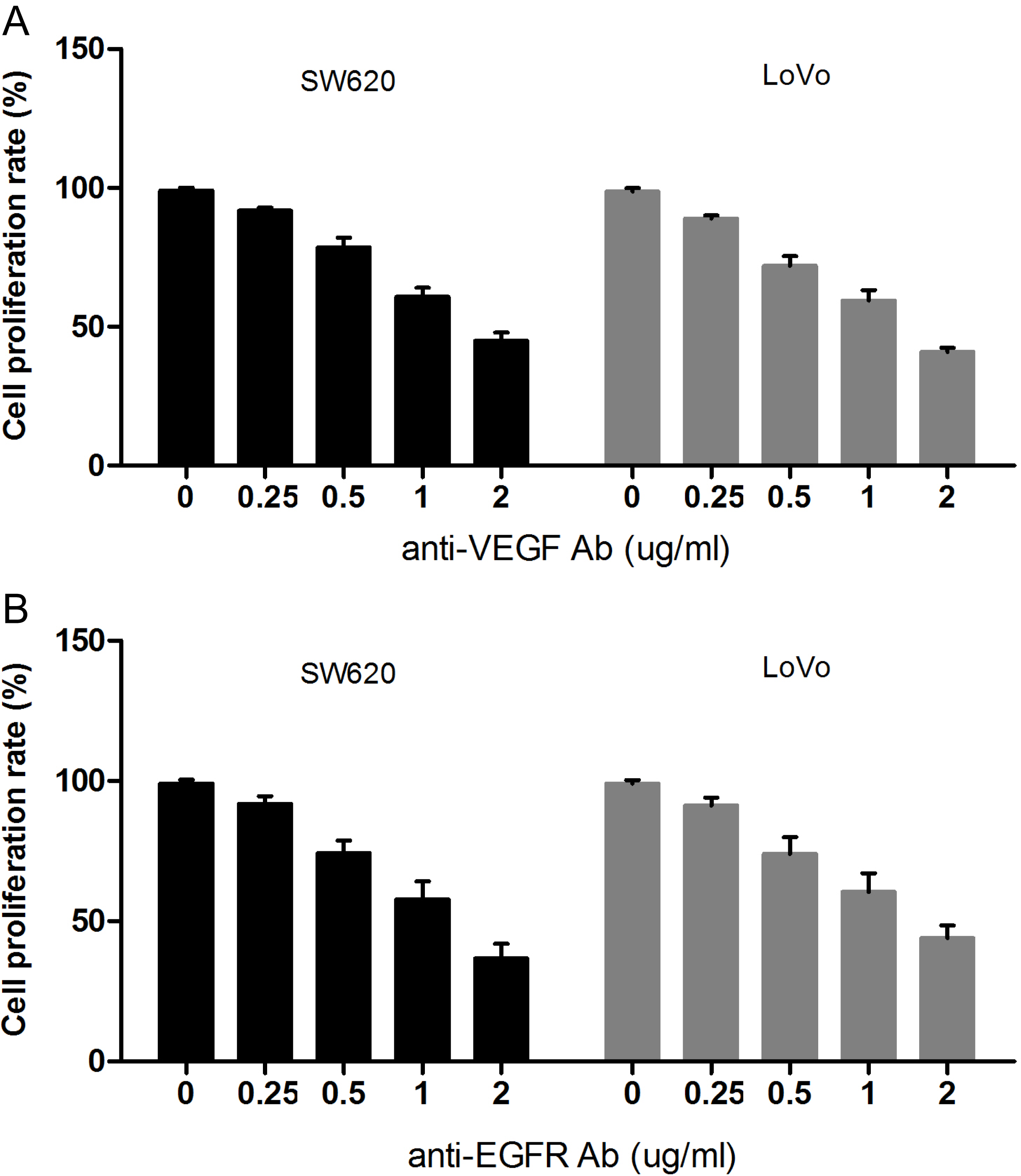

Supplement: Additional file 1: Figure S1. — Effect of different anti-VEGF mAb or anti-EGFR mAb concentration on the proliferation of SW620 and LoVo cells in vitro. A The proliferation rate of SW620 and LoVo cells were analyzed by CCK-8 assay in different anti-VEGF mAb concentration. B The proliferation rate of SW620 and LoVo cells were analyzed by CCK-8 assay in different anti-EGFR mAb concentration. (TIF 1121 kb) [file 12885_2016_2834_MOESM1_ESM.tif]

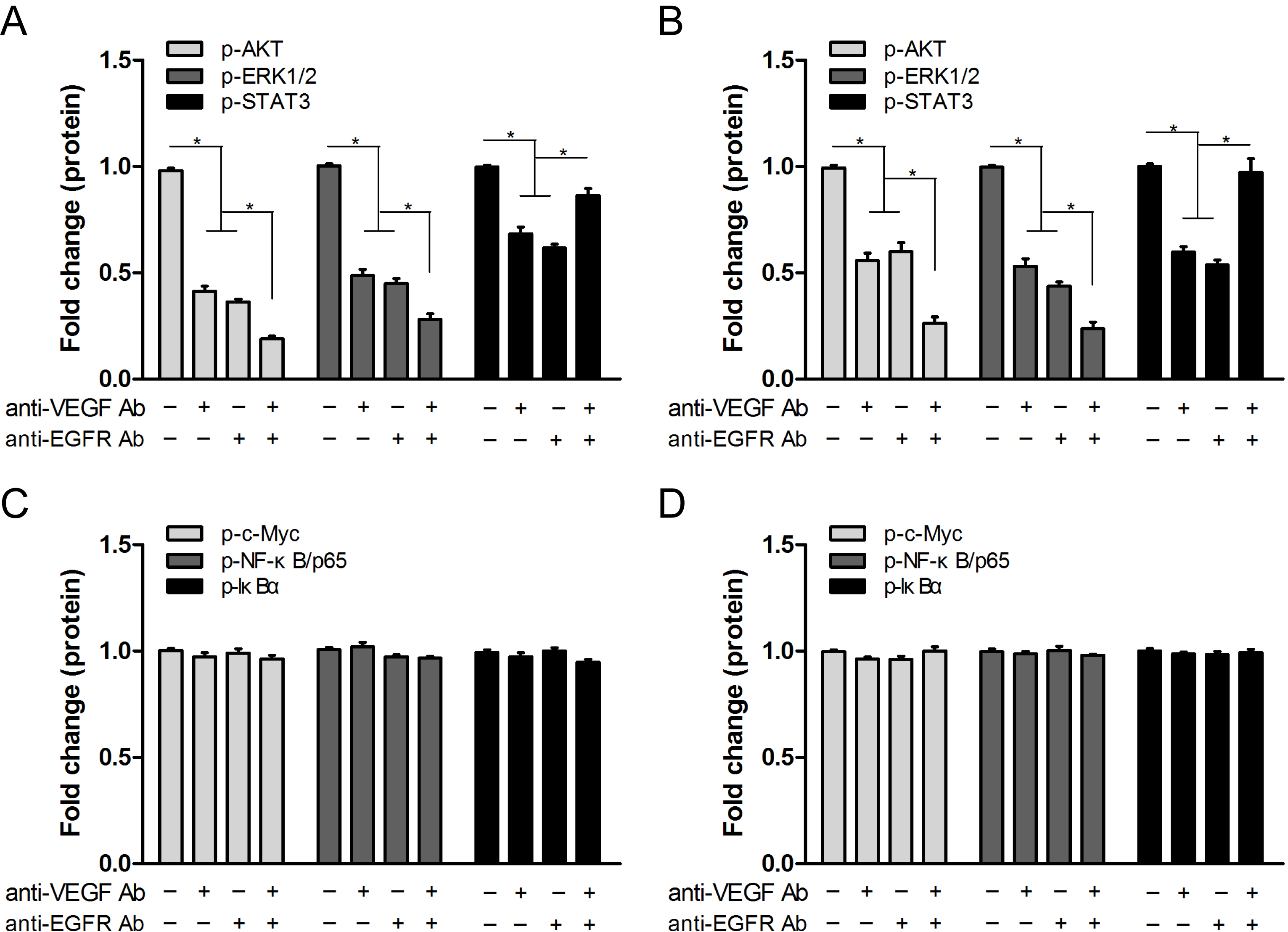

Supplement: Additional file 2: Figure S2. — Combined application of anti-VEGF and anti-EGFR antibodies inhibits AKT and ERK signaling pathways in mice model. A The expression of p-AKT, p-ERK1/2 and p-STAT3 were quantitatively analyzed in different SW620 cells tumors. B Western blot assay of different LoVo cells tumors (one clone, A). C The expression of p-c-Myc, p-NF-κB/p65 and p-IκBα were quantitatively analyzed in different SW620 cells tumors. D Western blot assay of different LoVo cells tumors (one clone, C). The data are representative of at least three different experiments ± SEM. *P < 0.05 (TIF 1285 kb) [file 12885_2016_2834_MOESM2_ESM.tif]

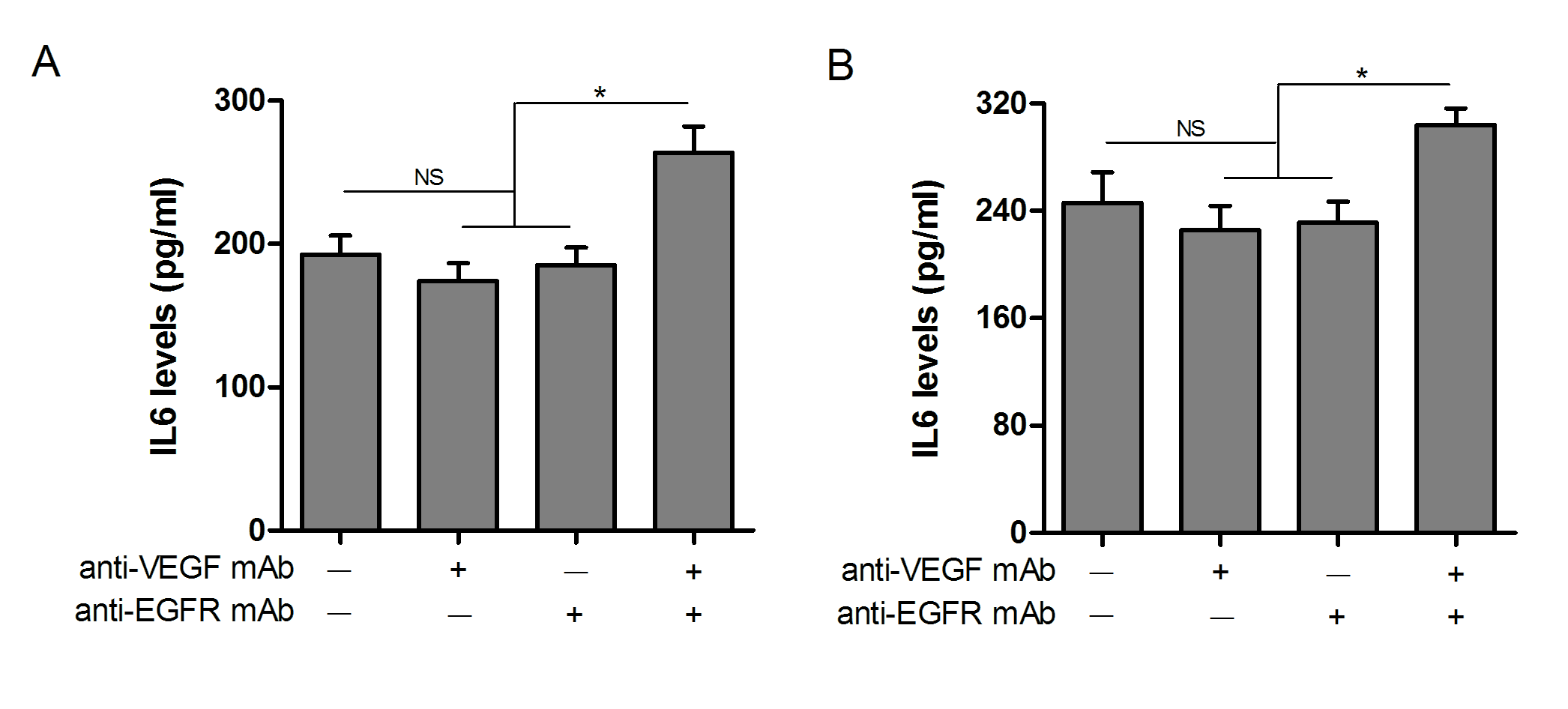

Supplement: Additional file 3: Figure S3. — The levels of IL6 in CRC cell tumors. A The expression of IL6 was quantitatively analyzed in different SW620 cells tumors. B ELISA assay of different LoVo cells tumors (one clone, A). The data are representative of at least three different experiments ± SEM. NS: No statistical significance; *P < 0.05 (TIF 1783 kb) [file 12885_2016_2834_MOESM3_ESM.tif]
